# Supplementary material for: Layer-by-Layer Electrode Fabrication for Improved Performance of Porous Polyimide-Based Supercapacitors
Source: Materials (Basel). 2021 Dec 21;15(1):4. doi: 10.3390/ma15010004 (PMC8745899; doi:10.3390/ma15010004)
Supplement: Supplementary file 1 [file materials-15-00004-s001.zip › materials-1513148-supplementary.pdf]

# Layer-by-Layer Electrode Fabrication for Improved Performance of Porous Polyimide-Based Supercapacitors

**Niranjala Fernando [1,†], Hugo Veldhuizen [2,†], Atsushi Nagai [3], Sybrand Van der Zwaag [2] and Amor Abdelkader [1,*]**

[1] Department of Engineering, Talbot Campus, Bournemouth University, Fern Barrow, Poole BH12 5BB, UK; nweerahannadige@bournemouth.ac.uk
[2] Faculty of Aerospace Engineering, Delft University of Technology, Kluyverweg 1, 2629 Delft,
The Netherlands; h.v.veldhuizen@tudelft.nl (H.V.); s.vanderzwaag-1@tudelft.nl (S.V.d.Z.)
[3] Department of Electrical and Electronic Information Engineering, Toyohashi University of Technology, Hibarigaoka-1-1 Tenpakucho, Toyohashi 441-8580, Japan; nagai.atsushi.rn@tut.jp
* Correspondence: aabdelkader@bournemouth.ac.uk
† These authors contributed equally to this work.

## Supporting information

Imide-bond formation after the polymerisation was confirmed with FT-IR spectroscopy (Figure S1). The characteristic N-H stretching vibrations of the amine-containing monomer TAPB around 3400 cm$^{-1}$ and the anhydride carbonyl vibrations of the PTCDA monomer (1730 and 1754 cm-$^{1}$) were not observed for **Per-TAPB-PPI**. Imide carbonyl vibrations were observed in the synthesised porous polymer: at 1665 and 1701 cm$^{-1}$. Lastly, the broad peak at 1344 cm$^{-1}$ was attributed to imide C─N stretching. These results indicate the successful conversion of the monomers into the polyimide polymer.

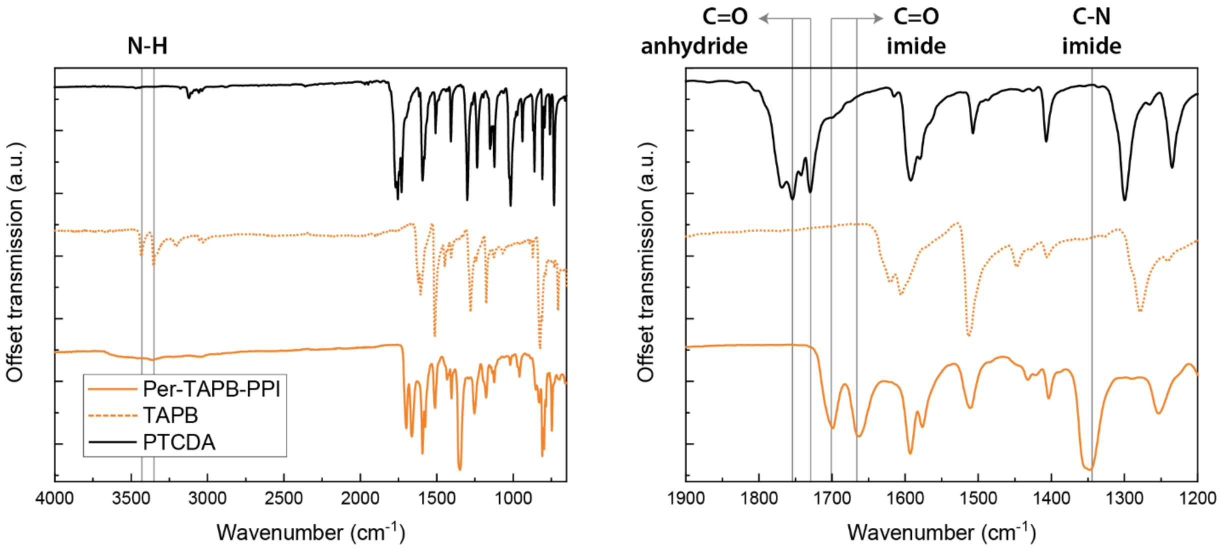

**Figure S1** FT-IR spectra of **Per-TAPB-PPI** and its originating monomers PTCDA and TAPB.

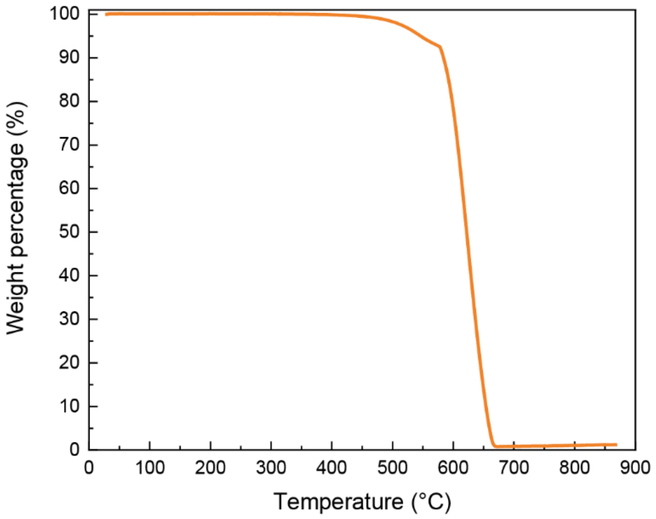


**Figure S2** TGA profile of **Per-TAPB-PPI** heated from 30 to 860 °C at a rate of 10 °C / min under constant flow of nitrogen gas.

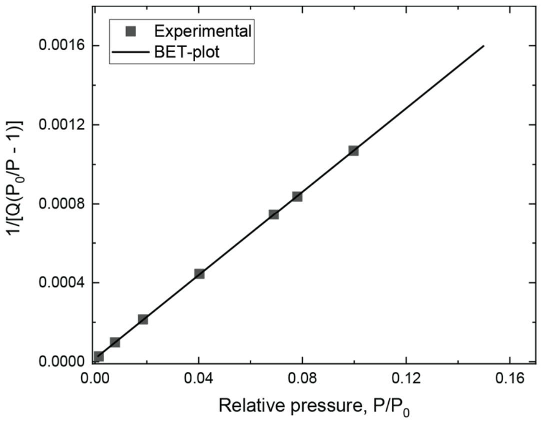


| BET Report | |
|---|---|
| | |
| BET surface area: | 411.5200 ± 1.3454 m²/g |
| Slope: | 0.010561 ± 0.000035 g/cm³ STP |
| Y-intercept: | 0.000016 ± 0.000002 g/cm³ STP |
| C: | 663.972094 |
| Qm: | 94.5463 cm³/g STP |
| Correlation coefficient: | 0.9999733 |
| Molecular cross-sectional area: | 0.1620 nm² |

**Figure S3** BET plot of **Per-TAPB-PPI**.

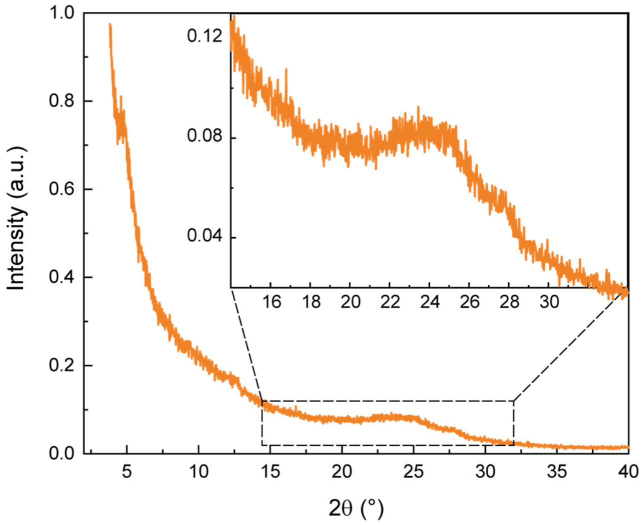


**Figure S4** PXRD pattern of **Per-TAPB-PPI**.

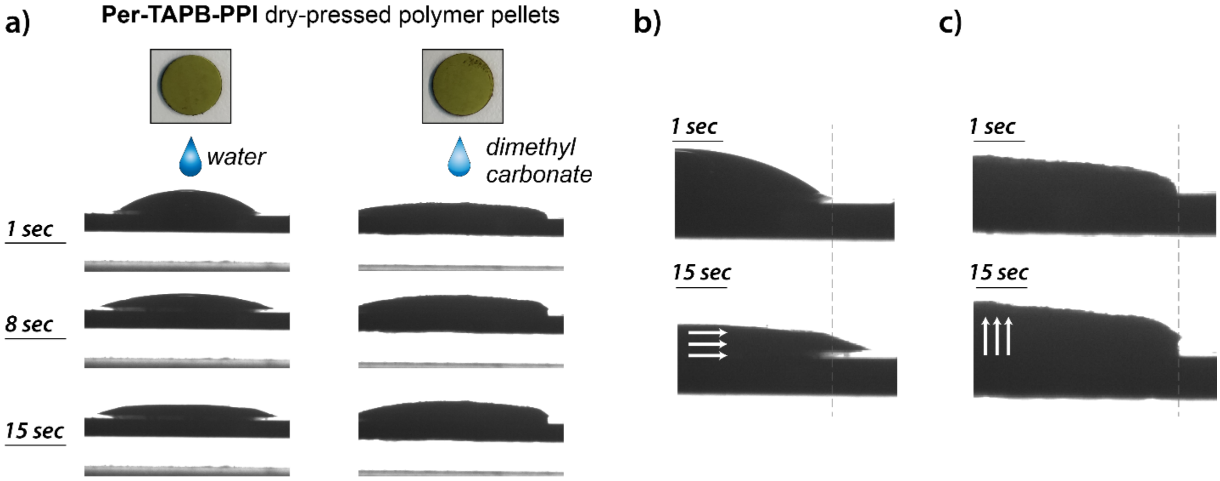


**Figure S5** Contact angle measurements of dry-pressed **Per-TAPB-PPI** pellets with either dimethyl carbonate or water as liquid. Images were taken after 1, 8, and 15 seconds. b) Zoom-in of the 1 sec and 15 sec photographs regarding the water experiment showing droplet spreading. c) Zoom-in of the 1 sec and 15 sec photographs regarding the dimethyl carbonate experiment showing swelling of the pellet.

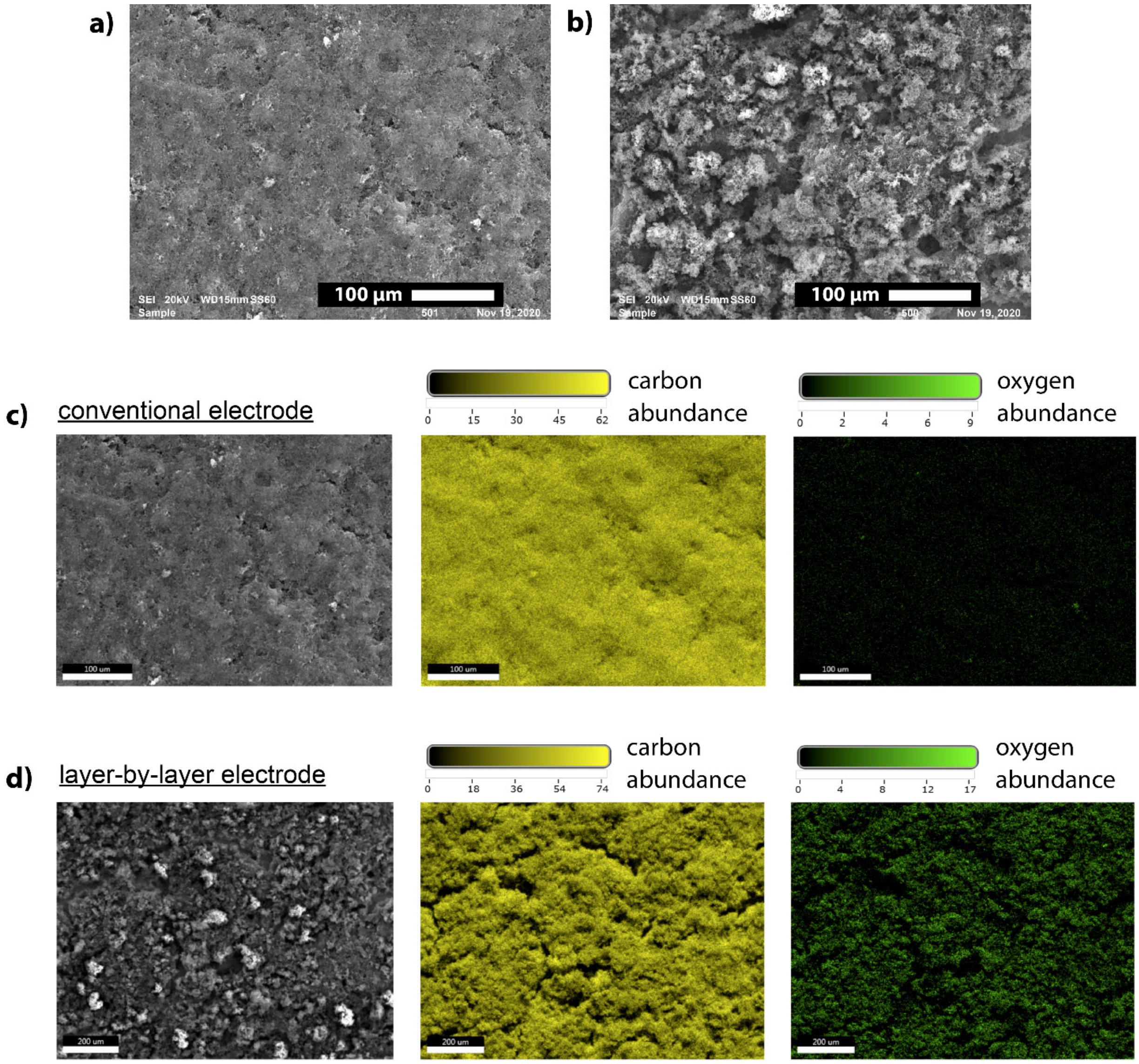


**Figure S6 a)** SEM micrograph of the surface of a **Per-TAPB-PPI**-based electrode prepared by a traditional mixing method. **b)** SEM micrograph of the surface of a **Per-TAPB-PPI**-based electrode prepared by a layer-by-layer method. **c)** SEM micrograph of the surface of a **Per-TAPB-PPI**-based electrode prepared by a traditional mixing method, including EDX mapping filtering for carbon and oxygen. **d)** SEM micrograph of the surface of a **Per-TAPB-PPI**-based electrode prepared by a layer-by-layer method, including EDX mapping filtering for carbon and oxygen.

**Table S1** Comparison of our supercapacitor systems with previously reported COF based electrodes.

| Electrode material | Electrolyte | Current density | Specific capacitance (F $g^{-1}$) | Capacitance retention %/ cycles | Refs. |
|---|---|---|---|---|---|
| **TPDA-1** | 1 M $H_2SO_4$ | 1 A $g^{-1}$ | 348 | 95/1000 | S1 |
| **DAAQ-TFP COF** | 1 M $H_2SO_4$ | 0.1 A $g^{-1}$ | 48±10 | 79/ 5000 | S2 |
| **TFP-TPA COF** | 1 M KOH | 2 A $g^{-1}$ | 291.1 | 91/ 5000 | S3 |
| **TFP-TPP COF** | 1 M KOH | 2 A $g^{-1}$ | 185.5 | 88.2/ 5000 | S3 |
| **TFP-Car COF** | 1 M KOH | 2 A $g^{-1}$ | 149.3 | 90.4/ 5000 | S3 |
| **TaPa-Py COF** | 1 M $H_2SO_4$ | 0.5 A $g^{-1}$ | 209 | 92/ 6000 | S4 |
| **DAB-TFP COF** | 1 M $H_2SO_4$ | 0.5 A $g^{-1}$ | 98 | - | S4 |
| **BIBDZ** | 1 M $H_3PO_4$ | 0.5 A $g^{-1}$ | 88.4 | 93.61/ 5000 | S5 |
| **NWNU-COF-1** | 6 M KOH | 0.25 A $g^{-1}$ | 155.38 | 100/ 20000 | S6 |
| **An-CPOP-2** | 1 M KOH | 0.5 A $g^{-1}$ | 98.4 | 95.3/ 2000 | S7 |
| **TPT-DAHQ** | 1 M KOH | 0.5 A $g^{-1}$ | 256 | 98.8/ 1850 | S8 |
| **Per-TAPB-PPI layered** | 1 M LiPF6 | 0.4 A $g^{-1}$ | 388 | 90.6 / 5000 | This work |
| **Per-TAPB-PPI traditional** | 1 M LiPF6 | 0.1 A $g^{-1}$ | 178 | 93.9 / 5000 | This work |


1. P. Bhanja, S. K. Das, K. Bhunia, D. Pradhan, T. Hayashi, Y. Hijikata, S. Irle, A. Bhaumik, ACS Sustainable Chem. Eng. 2018, 6, 202–209.

2. C.R. DeBlase, K.E. Silberstein, T.-.T. Truong, H.D. Abruña, W.R. Dichtel β-ketoenamine-linked covalent organic frameworks capable of pseudocapacitive energy storage, J. Am. Chem. Soc. 2013, 135, 45, 16821–16824.

3. EL-Mahdy, A.F., Hung, Y.H., Mansoure, T.H., Yu, H.H., Hsu, Y.S., Wu, K.C. and Kuo, S.W., 2019. Synthesis of [3+ 3] β-ketoenamine-tethered covalent organic frameworks (COFs) for high-performance supercapacitance and CO2 storage. Journal of the Taiwan Institute of Chemical Engineers, 103, pp.199-208.

4. Khattak, A.M., Ghazi, Z.A., Liang, B., Khan, N.A., Iqbal, A., Li, L. and Tang, Z., 2016. A redox-active 2D covalent organic framework with pyridine moieties capable of faradaic energy storage. Journal of Materials Chemistry A, 4(42), pp.16312-16317.

5. Roy, A., Mondal, S., Halder, A., Banerjee, A., Ghoshal, D., Paul, A. and Malik, S., 2017. Benzimidazole linked arylimide based covalent organic framework as gas adsorbing and electrode materials for supercapacitor application. European Polymer Journal, 93, pp.448-457.

6. Xue, R., Guo, H., Yue, L., Wang, T., Wang, M., Li, Q., Liu, H. and Yang, W., 2018. Preparation and energy storage application of a long-life and high rate performance pseudocapacitive COF material linked with–NH–bonds. New Journal of Chemistry, 42(16), pp.13726-13731.

7. Mohamed, M.G., Zhang, X., Mansoure, T.H., El-Mahdy, A.F., Huang, C.F., Danko, M., Xin, Z. and Kuo, S.W., 2020. Hypercrosslinked porous organic polymers based on tetraphenylanthraquinone for CO2 uptake and high-performance supercapacitor. Polymer, 205, p.122857.

8. El-Mahdy, A.F., Hung, Y.H., Mansoure, T.H., Yu, H.H., Chen, T. and Kuo, S.W., 2019. A Hollow Microtubular Triazine-and Benzobisoxazole-Based Covalent Organic Framework Presenting Sponge-Like Shells That Functions as a High-Performance Supercapacitor. Chemistry–An Asian Journal, 14(9), pp.1429-1435.
